# Re-Assembly and Analysis of an Ancient Variola Virus Genome

**DOI:** 10.3390/v9090253

**Published:** 2017-09-08

**Authors:** Chad Smithson, Jacob Imbery, Chris Upton

**Affiliations:** Department of Biochemistry and Microbiology, University of Victoria, Victoria, BC V8W 3P6, Canada; chadsmiths83@gmail.com (C.S.); jimbery@live.ca (J.I.)

**Keywords:** variola virus, cowpox, orthopoxvirus, virus virulence, phylogeny, smallpox, camelpox, taterapox, poxvirus

## Abstract

We report a major improvement to the assembly of published short read sequencing data from an ancient variola virus (VARV) genome by the removal of contig-capping sequencing tags and manual searches for gap-spanning reads. The new assembly, together with camelpox and taterapox genomes, permitted new dates to be calculated for the last common ancestor of all VARV genomes. The analysis of recently sequenced VARV-like cowpox virus genomes showed that single nucleotide polymorphisms (SNPs) and amino acid changes in the vaccinia virus (VACV)-Cop-O1L ortholog, predicted to be associated with VARV host specificity and virulence, were introduced into the lineage before the divergence of these viruses. A comparison of the ancient and modern VARV genome sequences also revealed a measurable drift towards adenine + thymine (A + T) richness.

## 1. Introduction

Smallpox, which is caused by the poxvirus variola virus (VARV) [1], was declared eradicated almost 40 years ago, following a massive worldwide vaccination campaign [2,3]. The disease may have been the world’s worst scourge, with reports that more than 500 million were killed during the Twentieth Century alone [4]. VARV and vaccinia virus (VACV), which was used as a live vaccine, belong to the *Orthopoxvirus* genus within the *Poxviridae* family and have linear dsDNA genomes of approximately 200 kb. VACV is the most studied poxvirus and serves as a model virus for the family. However, several other Orthopoxviruses are notable: monkeypox viruses (MPXV) [5] are zoonotic; ectromelia viruses (ECTV) [6] have served as a model for smallpox in mice; rabbitpox viruses [7,8] are VACV variants that are hypervirulent in rabbits; and cowpox viruses [9,10,11], which despite their names, actually form several species, encode the most genes.

More than 300 essentially complete genomes have been sequenced from poxviruses (*Chordopoxvirinae* subfamily) that infect vertebrates. Although not all are yet officially recognized, these likely fall into at least 18 genera. When these viruses are aligned, the highest synteny and DNA sequence identity is found within a central core region of approximately 80 to 100 kb, with a gene density averaging approximately one per kb. Outside of this core, the genes, which mostly encode host range and virulence factors and are not present in all viruses, are frequently rearranged and have significantly lower levels of DNA and amino acid (aa) conservation than genes in the core region. Thus, when different sets of genes are compared for a given group of viruses, it is not uncommon to observe significantly different levels of conservation because core genes are more highly conserved [12].

Several other factors complicate genome alignments and the ubiquitous phylogenetic trees that are used to display the evolutionary relationships between the viruses. First, when complete genomes are aligned, the alignment software is frequently confused by the presence of large insertions/deletions (indels) or gene rearrangements in conjunction with the variety of paralogs present outside the central core. The alignment of paralogs instead of orthologs may lead to serious errors in the trees. Second, numerous examples of homologous recombination, which go against the underlying assumptions of phylogenetics, have been described within poxvirus genomes [9,13,14,15].

In previous work [16], we hypothesized that the limited host range (only humans) and severe virulence might be linked to a relatively large number of single nucleotide polymorphisms (SNPs) grouped together in the VARV ortholog of the *VACV-Cop-O1L* gene (membrane protein) and that a recombination event had created this sequence arrangement. Therefore, it was of great interest when the sequencing of a VARV isolated from a mummy, which was estimated to be approximately 350 years old, was announced [1]. This genome (GenBank KY358055.1) was described, in the supplementary material, as consisting of 31 contigs with padding of Ns (unidentified nucleotides) to allow alignment to a reference sequence (VARV-India-1967; National Center for Biotechnology Information (NCBI) Reference Sequence NC_001611.1). However, when the sequence file was examined, there were 121 blocks of Ns ranging in size from one to 626 nucleotides (nt). The shorter blocks of Ns (46 are shorter than 10 nt), which are frequently close together, may represent unresolved nucleotide calls rather than all being spacers between un-joined contigs. Here we describe significant improvements to the assembly of the reported ancient VARV genome sequence data [1] and further characterization of the evolution of VARV.

## 2. Materials and Methods

### 2.1. Retrieval of Genome Sequences and Alignment

The genomes used for the construction of multiple sequence alignments (MSAs) are listed in Table 1. The sequences were aligned using MAFFT [17], and both ends of the genome were removed, leaving a 98 kb core region spanning the VACV-WR genome 32958–134689 nt, from gene *VACV-WR-043* (ribonucleotide reductase, small subunit) to *VACV-WR-144* (RNA polymerase (RPO132)). The sequences were then manually edited using Base-By-Base (BBB; [18]) software to correct alignment errors produced by the software. All alignment columns containing gap-characters were removed prior to the construction of the phylogenetic trees.

### 2.2. Visual Examination of the MSA

The BBB software highlights SNPs in several ways. The user can choose to compare sequences against the top sequence of the alignment, against the consensus sequence, or in a pairwise fashion. Differences between SNPs are highlighted by blue blocks; insertions and deletions are shown by green and red blocks, respectively. This highlighting makes otherwise unrecognizable short patterns of SNPs easily visible, allowing the user to scan the sequence alignment visually for small and imperfect patterns of SNPs and indels. Although this process sounds archaic, the human eye/brain is still usually most effective when looking for novel patterns in subsets of the sequences in MSAs.

### 2.3. Phylogenetic Tree Construction and Evolutionary Analyses

Phylogenetic analyses were performed using a 98 kb alignment of the conserved core region of orthopoxviruses between *VACV-Cop-F4L* and *Cop-A24R* orthologs with any gap-containing columns removed. Maximum-likelihood trees were constructed using RAxML (Randomized Axelerated Maximum Likelihood) [19] under the GTRGAMMA (General time reversible) base substitution model using 1000 bootstrap replicates. We performed an evolutionary analysis of the VARV species using the Bayesian Markov chain Monte Carlo (MCMC) inference methods with BEAST 2 (Bayesian evolutionary analysis by sampling trees) software [20], with the rooted RAxML constructed maximum-likelihood tree as the starting tree. All of the viruses included in the evolutionary analysis had reliable collection dates, with the exception of VARV-VD21-uvic (our assembly), which was previously dated using radiocarbon dating techniques [1] , and VARV-V1588 and VARV-V563, which were previously dated by the degree of d- and l-aspartic acid racemization [21]. BEAST 2 was run using a fixed clock model, constant population size coalescent priors, and a 100,000,000 MCMC chain length. The maximum clade credibility tree produced was generated under a GTR substitution model using gamma and invariant sites.

## 3. Results

### 3.1. Re-Assembly of the VARV-VD21 Genome

**Figure 1:**
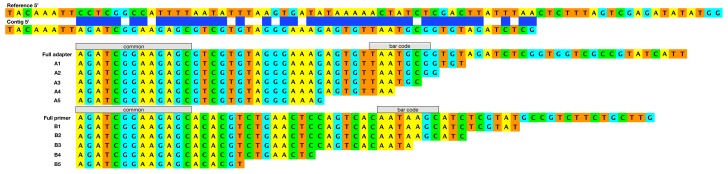
Examples of the Illumina adapter and primer fragments found attached to terminated contigs. The sequences are displayed 5′ > 3′. The top two sequences represent the reference variola virus (VARV) genome with the end of a contig immediately below it. At the left, the reference and contig match perfectly; the blue boxes show a region of mostly mismatching single nucleotide polymorphisms (SNPs) due to the adapter, which terminates the contig. The full adapter and primer sequences are shown with examples of fragments of adapters (A1–A5) and fragments of primers (B1–B5) found to terminate contigs.

The previously reported VARV-VD21 genome of 31 contigs was created using de novo and reference mapped assemblies. However, the GenBank file (KY358055.1) of this ancient genome contains 121 groups of Ns, which range in size from 1 to 626 nt and total 3,748 nt. Therefore, before attempting to search for evidence of recombination in the severely fragmented genome, we set out to try and fill some of the gaps in the published sequence by using alternative assembly strategies. Our first attempts at assembling the data provided by the Sequence Read Archive (SRP091673), using Cutadapt [22] to trim Illumina adapters and MIRA (Mimicking Intelligent Read Assembly) [23] and SPAdes (St. Petersburg genome Assembler) [24] for assembly, created 56 contigs that could not be resolved by modifying the assembly parameters. However, when these contigs were viewed and aligned to the same reference sequence (VARV-India-1967) using BBB, it was obvious that the ends of the contigs were effectively terminated by reads that contained variants of the sequencing adapters normally removed by the Cutadapt module (Figure 1).

**Figure 2:**
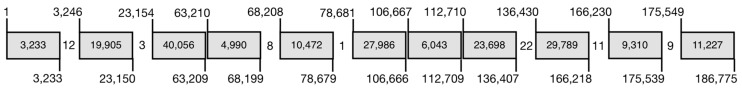
The grey boxes show the contig sizes of reassembled VARV-VD21-uvic. The top-left and bottom-right numbers indicate the start and stop positions of the contigs, respectively. Numbers between the grey boxes show the number of Ns needed to align the contigs with the VARV-Bang-1975 reference genome. Some contigs abut but do not have reads to join them.

Therefore, we repeated the trimming process using Cutadapt a further three times, modifying the adapter information to match the remaining adapters at each step. This process reduced the number of contigs to 11, with gaps ranging from 0 to 22 nt when aligned to a reference genome; contigs with gaps of 0 nt abut but do not overlap. Even searches through the fastq files for specific nucleotide sequences within reads failed to find reads that could join these final contigs. Figure 2 displays the organization of our final assembly after alignment with VARV-Bang-1975. The majority of improvement in assembly and coverage came from the repeated removal of the fragmented Illumina adapters. However, we also accepted some very low coverage regions that were 100% consistent with a consensus built from all known VARV genome sequences. Following this reassembly step, these new contigs were compared to the published VARV-VD21 sequence and all differences were examined manually using TABLET software [25]. Surprisingly, of 79 nt differences between these two sequences, all but 10 had been called wrong by the SPAdes software, probably due to failed mismatch correction in regions of low coverage. Next, the new VARV-VD21 contigs were compared to previously sequenced VARV genomes, and nucleotides unique to this new genome were checked for correct nt calling with TABLET; all these SNPs were correct. Finally, we aligned the contigs with VARV-Bang75 as a reference (see below) and padded the contigs with Ns to create an alignable genome sequence. A comparison of our new assembly to the published VARV-VD21 sequence revealed that the number of contigs was reduced from 122 to 11 and that the total number of Ns was reduced from 3748 to 66; this revised version of the ancient VARV genome is denoted as VARV-VD21-uvic and is used in all subsequent analyses. This new assembly has been submitted to GenBank (Accession number BK010317).

**Figure 3:**
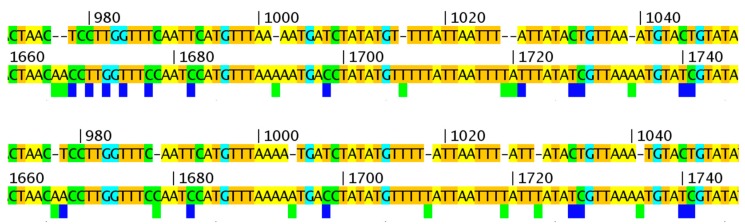
Optimization of alignments with multiple small gaps. Upper/lower panels: top sequence: VARV-India-1967, bottom sequence: VARV consensus. Top panel: MAFFT global genome alignment with default parameters. Bottom panel: manually corrected alignment with reduced SNPs. The blue and green boxes indicate SNPs and gaps in the VARV-India-1967 sequences, respectively. Numbers refer to position in 98 kb alignment.

**Figure 4:**
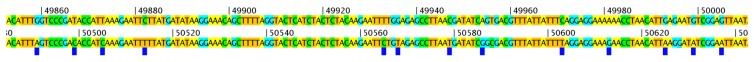
“Unique” VARV sequence matches VACV genomes. The top sequence is from VARV-India-1967 and bottom sequence is from VARV-BGD75. The blue boxes show SNPs, which are unique to VARV-India-1967 among all VARVs. All of these SNPs are found in VACV sequences. Numbers refer to position in 98 kb alignment.

For any comparative genomic analysis to be meaningful, it is essential the sequences that are aligned are both (1) optimally aligned and (2) related through common ancestry. As noted above, the organization of genes outside of the central core is such that large indels and paralogous genes often create alignment errors. Therefore, we processed the alignments that are used below in several ways to minimize alignment errors; some of these steps were included to make the data easier to view and interpret. First, we built a MAFFT MSA [17] of all previously published VARV whole genomes. Viewing of this alignment confirmed not only that the terminal regions of the VARV are the most variable but also that (1) it is impossible to create a meaningful alignment along the full length of all the VARV genomes, (2) default MAFFT parameters, set for large DNA sequences, fail to correctly align short regions that need multiple small indels, and (3) the vast majority of repeat regions and indels are within the 25 kb terminal regions. Interestingly, the first region we noticed that was misaligned was a region in VARV-India-1967, which was used as the reference strain by Duggan et al. Figure 3 illustrates a region unique to VARV-India-1967, as aligned by MAFFT to a consensus of all other VARV genomes. However, this alignment can be significantly improved, reducing 12 SNPs to seven by the redistribution of the seven gapped positions. Although the new alignment has seven independent gaps instead of two gaps of 2 nt and three gaps of 1 nt, this arrangement places every one of the seven gaps opposite a short homopolymer run, which strongly suggests that at least some of these might be sequencing errors in the VARV-India-1967 sequence. Therefore, we scanned the other SNPs that were unique to VARV-India-1967 for unusual features. In the short region, 49,859 to 50,005 (146 nt), we found a cluster of 13 such SNPs; a search of the database of poxvirus sequences revealed that this apparently unique VARV region from India-1967 matches perfectly to an orthologous region in a series of VACV isolates (Figure 4). This result suggests further errors in the sequencing process of VARV-India-1967 or a recombination event between an ancestor of this virus and possibly a vaccinia virus (vaccine) isolate. In view of these results, we decided to select an almost gap-free central region of approximately 98 kb that could be checked for regions of misalignment for further phylogenetic analysis. In addition, we selected subsets of the available genomes, representing the most divergent of the completely sequenced VARV, cowpox (CPXV), and VACV groups and also included camelpox (CMLV) and taterapox (TATV) genomes. After simplifying the MSA by removing any nt column that contained an N (from VARV-VD21-uvic) and also any nt column that contained a gap character (in any genome), we compared VARV-VD21-uvic to the other VARV sequences. Within this region of the alignment, the 10 VARV-major strains had a range of 223 to 254 nt differences to VARV-VD21-uvic (mean 239), whereas VARV-India-1967 had 298 nt differences (>3 standard deviations above the mean). Given this result and the previous anomalies with VARV-India-1967 observed here and by others [26], we decided to exclude this genome from further phylogenetic analyses.

### 3.2. Phylogenetic History of VARV

**Figure 5:**
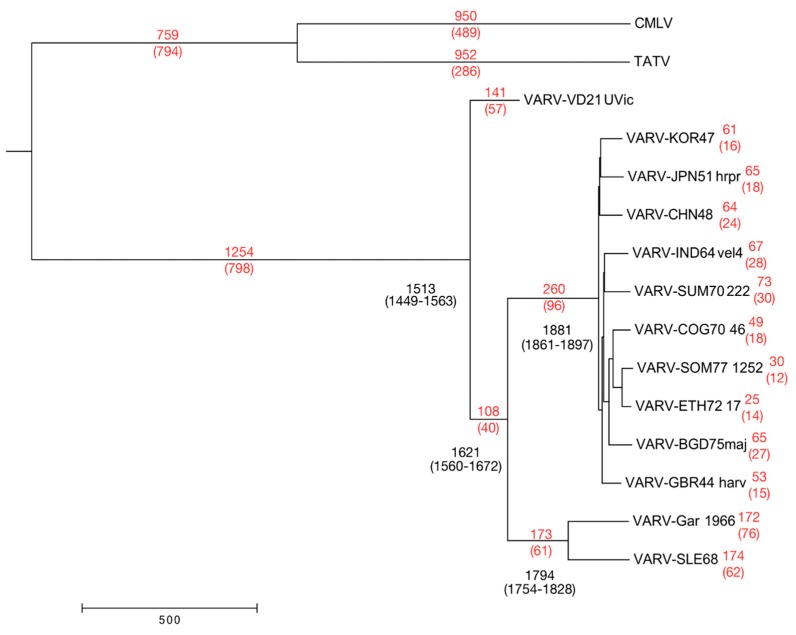
Maximum clade credibility tree depicting the evolutionary history of VARV, CMLV, and TATV species. The VARV, CMLV, and TATV genomes were aligned with MAFFT and cropped to the poxvirus core region between the orthologs of VACV-Cop-F4L and VACV-Cop-A24R, and any columns containing a gap were removed. The median estimated tMRCA for each strain is adjacent to major nodes of the tree, with the 95% higher posterior density (HPD) interval, the range covering 95% of all estimates, below. The time since the most recent divergence event as well as the number of unique SNPs (in brackets) is reported (red) for each branch. The scale bar is in years.

As in the analysis performed by Duggan et al. we constructed an evolutionary time tree comparing the core regions of CMLV, TATV, and VARV strains (Figure 5). However, the input data was somewhat different in that we (1) used a more complete version of the VARV-VD21 genome (VARV-VD21-uvic), (2) used a manually corrected alignment, (3) added CMLV and TATV genomes, (4) omitted VARV-India-1967, and (5) added a VARV-major genome from 1942 and a VARV-minor genome from 1850. The tree depicts the evolutionary divergence of the VARV species and provides estimates of the “time since their most recent common ancestor” (tMRCA), where the most recent common ancestor is the latest state of the ancestral virus from which each member of the group of viruses has directly descended. The dates depicted at the nodes of the tree represent the median date estimated from a Bayesian evolutionary analysis performed using BEAST 2 under a fixed clock model and using constant size coalescent priors as per the analysis performed by Duggan et al. [1]. The inclusion of CMLV and TATV sequences provides a stronger root to the rest of the VARV sequences for the estimation of the tMRCAs, as well as providing an estimate of when these orthopox viruses diverged into distinct species and perhaps became host specific.

The most significant difference between the data presented in Figure 5 and the previous work is that our results push back the date of the tMRCA by about 100 years for all VARVs (from 1588–1645 to 1470–1563), as well as the split of the VARV major and minor clades (from 1734–1793 to 1579–1667). Interestingly, the dates for speciation of all VARV and the major/minor species are midway between those calculated by Duggan et al. [1] and those recently calculated by Pajer et al. [21], who sequenced the VARV-major genome from 1942 and the VARV-minor genome from 1850. The number of unique SNPs associated with particular viruses at nodes on the tree (Figure 5) are consistent for all of the VARVs, suggesting that, after the speciation of VARV, there has been little to no recombination with other poxvirus species.

### 3.3. Drift in Nucleotide Composition

The nucleotide composition of poxvirus genomes is an interesting puzzle in that the A + T% varies between 33% (squirrelpox virus) and 82% (amsacta moorei entomopoxvirus), but the mechanism by which the drift occurs is unknown. Given that VARV-VD21-uvic represents a true ancient genome, we decided to determine if a difference in the A + T% could be detected between the ancient and modern VARV genomes. Using the same central core of 98 kb, BBB calculated the A + T% for the ancient and modern sequences to be 67.0 and 67.1, respectively. The plot nucleotide content function of BBB showed no discernable difference but emphasized that the A + T composition within a genome varies by more than 10% if a sliding window of 1 kb is plotted across the genome core. However, when we focused on each of the individual SNPs, comparing ancient to modern nucleotides, we found that approximately twice as many nt changes were guanine/cytosine (G/C) to A/T (157 nt) than were A/T to G/C (79 nt; Table 2). This same dominance of G/C to A/T SNPs was found when comparing the VARV genome isolated in 1850 to the modern VARVs. Thus, the core of the VARV genome has increased in A + T richness by approximately 0.08% in about 300 years. Interestingly, although the CMLV core is slightly less A + T rich than the VARV core, when unique SNPs in CMLV are compared to a consensus of VARV, VACV, TATV, and CPXV-humAac09, there are four times as many SNPs that change G/C to A/T than that change A/T to G/C (Table 3).

### 3.4. Evolution of the VARV Ortholog of VACV-Cop-O1L

**Figure 6:**
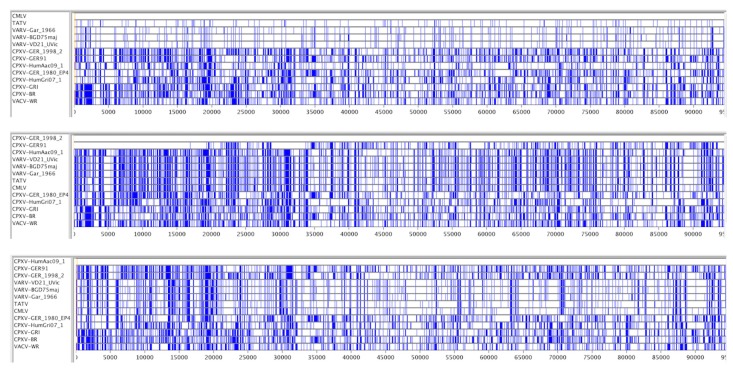
Base-By-Base (BBB) visual summaries of core alignments. SNPs unique to a single sequence have been converted to the consensus nucleotide, and gaps have been removed. Blue bars indicate a SNP; all sequences have been compared to the top sequence in each panel. Numbers refer to position in 98 kb alignment.

The key evolutionary events that lead to the creation of the human-specific pathogen VARV remain a mystery. However, in previous work, we suggested that one or more recombination events prior to the divergence of VARV, CMLV, and TATV produced a large number of SNPs in the ortholog of *VACV-O1L*, which has been identified as a virulence factor [27,28], may have been a significant factor [16]. Therefore, we examined the distribution of the SNPs associated with this gene in VARV-VD21-uvic and the recently sequenced CPXV-HumAac09, a representative of the VARV-like-CPXV clade [9]. First, a comparison of the VARV-VD21-uvic sequence with the modern *VARV-O1L* gene sequences found only six SNPs associated with changes distributed across the complete clades (major or minor) of VARV. Nucleotide changes 985R > K, 1029Silent and 1799A > V were found in all modern *VARV-O1L* gene sequences and nucleotide changes 775E > K, 1173Silent and 1942Y > H were found in the *VARV-major-O1L* gene sequences. When long sequences are used for phylogenetic analyses, the placement of the VARV-like-CPXVs and other CPXV clades on the phylogenetic tree is consistent. However, because of recombination events between these groups of viruses, it is important to remember that parts of the phylogenetic tree are not displaying true evolutionary relationships for all regions of the sequences. Furthermore, although the presence of orthopoxvirus recombination can be displayed on reticulated phylogenetic trees and with Bootscan analysis [9,29], these do not convey SNP detail about the relationships between the DNA sequences. Therefore, we used BBB to search for SNPs shared between particular sets of viruses and to provide a visual summary of the SNP patterns. The BBB visual summary (Figure 6) shows SNP differences in sequences compared with the top sequence in an MSA and also allows the user to change the scale of the display and re-order the sequences, both of which are very useful for discovering hidden relationships between the sequences. For example, in Figure 6, the top panel shows a mostly random distribution of SNPs among the VARV + TATV + CMLV sequences; the middle panel shows two large blocks of perfect identity between CPXVs GER-1998 and GER91 at the left side of the alignment; and the bottom panel compares CPXV-HumAac09 to the other sequences and shows a patchwork of regions in the various viruses that are identical to the VARV-like CPXV sequence.

**Figure 7:**
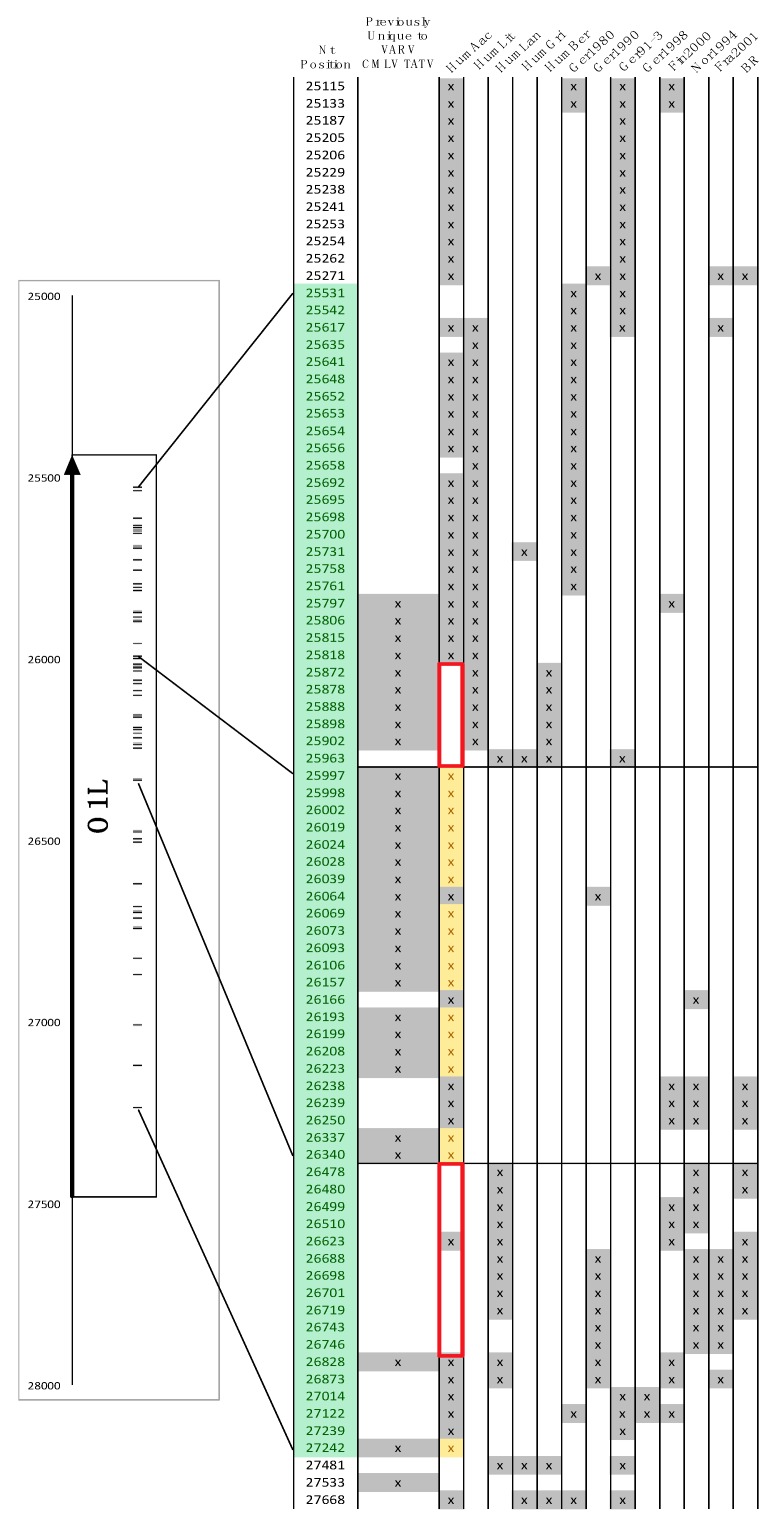
Organization of SNPs within the VARV O1L gene. Nucleotide positions refer to the gapped core alignment used previously. SNPs, shown by nucleotide position, were identified by the BBB search “find positions of nucleotides that are identical in VARV + CMLV + TATV, but different in all CPXVs and VACVs with a tolerance of 5” (i.e. up to five other genomes can match the VARV + CMLV + TATV nucleotide). SNPs in the “Previously Unique to VARV + CMLV + TATV” column are from previous work before the VARV-like CPXVs were sequenced. The positions that also match “tolerated CPXV genomes” are indicated by an “x” in columns labeled with CPXV strain. The arrow in left panel and SNPs shaded green indicate the extent of the *O1L* gene; yellow indicates SNPs that are unique to VARV + CMLV + TATV + VARV-like-CPXV sequences. Red boxes indicate regions where CPXV-HumAac09 does not match the VARV + CMLV + TATV nucleotide but where other CPXVs do.

**Figure 8:**
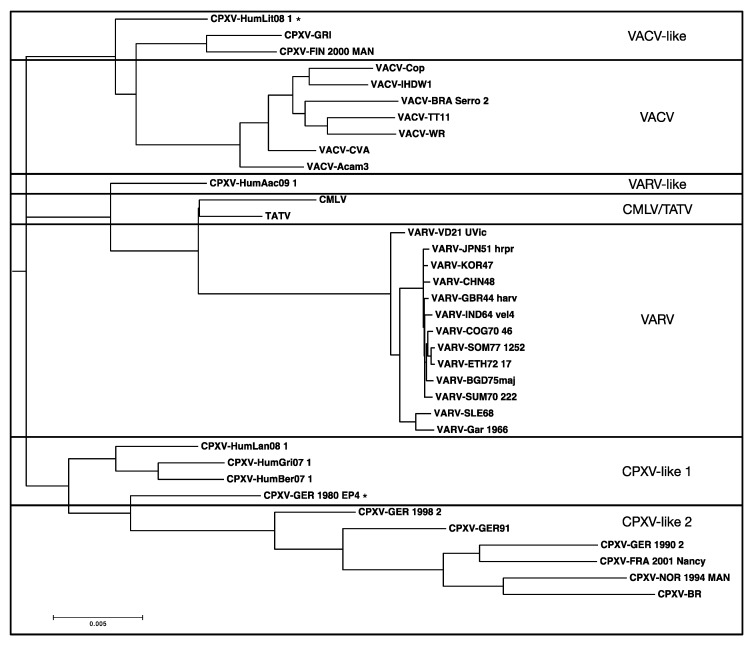
RAxML rooted phylogenetic tree. Species are delineated by bold lines; asterisks indicate isolates that do not fall into clear strain categories [9]. The tree was created from the MAFFT aligned orthopox core region (with gaps and Ns removed) using RAxML under a GTRGAMMA base substitution model using 1000 bootstrap replicates and visualized using MEGA7 (Molecular Evolutionary Genetics Analysis) software.

Previously, we found a block of SNPs in the *O1L* gene with the BBB search parameter “find position of nucleotides that are identical in VARV + CMLV + TATV but different in all CPXVs and VACVs” [16]. However, when this analysis was repeated with the new CPXVs included, no SNPs were found in *O1L* with the same query (Figure 7); a phylogenetic tree of these genomes is shown in Figure 8. This result indicates that the previously identified SNPs must be present in some of the new viruses, the most likely being CPXV-HumAac09, which represents the VARV-like-CPXVs. Therefore, we repeated the BBB query with the addition of a “*tolerance of 5*” parameter, which instructs BBB to allow up to five other sequences from the alignment to match the nucleotides otherwise unique to VARV + CMLV + TATV. BBB provides the position of the SNP and the number and names of the sequences falling into the set of “tolerated” matches (Figure 7). Most of the SNPs previously found to be unique to VARV + CMLV + TATV were found to be also present in CPXV-HumAac09 but not in any other CPXV strain (highlighted yellow in Figure 7). Another block of the original SNPs is subdivided into groups that are also in CPXV-HumAac09 + CPXV-HumLit, CPXV-HumAac09 + CPXV-HumLit + CPXV-Ger1980, or CPXV-HumLit + CPXV-HumBer. There are several other blocks of SNPs associated with VARV + CMLV + TATV, CPXV-HumAac09, and one to four other CPXVs in this particular alignment (Figure 7). Thus, the vast majority of the SNPs present in *O1L* and associated with the VARV clade of orthopoxviruses were introduced before the split of VARV + CMLV + TATV and the VARV-like-CPXVs, which can be represented here by CPXV-HumAac09 because they are almost identical. It is noteworthy that the *O1L* gene is nonfunctional in both TATV and CMLV but maintained in VARV genomes.

This unusual distribution of mutations within the O1L ortholog, which does not fit the phylogenetic tree, can be further demonstrated with a comparison of the concatenated protein sequences from the same central core (93 genes; *F4L-A24R*) of these viruses. The related BBB query “find positions of aa that are identical in VARV + CMLV + TATV + CPXV-Aac09 but different in all other CPXVs and VACVs” revealed a total of only 21 unique aa. However, six of these were concentrated within a 40 aa region of the O1L C-terminus.

## 4. Discussion

The history and evolution of variola virus is important because the devastating disease that this poxvirus causes has been instrumental in shaping human populations [4,26]. Although this virus has been eradicated from the global environment by the development and implementation of vaccination, there are several other poxviruses that, either naturally or as an epizootic, infect and cause disease in humans; these include molluscum contagiosum virus (MOCV) [30], monkeypox [5], V_014 [31], escaped VACV [32], orf [33], CPXV [9], and a novel orthopoxvirus isolated in Alaska [34]. Therefore, it is important to understand the viral mechanisms that control pathogenesis and host range. Based on a bioinformatics analysis of the orthopoxviruses, which found an unusual group of SNPs associated specifically with the VARV ortholog of *VACV-Cop-O1L*, we had previously hypothesized that this gene might have influenced the virulence and/or the host range of the evolving VARV clade of viruses. Curiously, this gene has been lost from CMLV and TATV, which also have very restricted host ranges and are the two most similar viruses to VARV.

It was therefore of great interest when several VARV genomes were sequenced from relatively ancient samples. Unfortunately, the genome sequence from the oldest virus, isolated from a mummified child approximately 360 years old, was reported as a GenBank file (Accession number: KY358055.1) with 3748 unknown nucleotides that broke the genome up into 122 segments. Before analyzing this highly fragmented DNA sequence, we attempted to improve the genome assembly and the quality of the sequence using the previously published sequence data. During this process, we discovered that our first assembly was similarly poor but observed that the contigs were in fact terminated by variant Illumina adapters that had not been removed from the sequencing reads by the Cutadapt software. Therefore, we repeated the assembly process with a series of adaptor sequences removed and followed up with a manual review of the assembly, accepting low coverage reads that agreed 100% with the consensus sequence from multiple VARV genomes. Thus, we arrived at a greatly improved genome sequence assembly that reduced the number of sequence segments from 122 to 11 and the total number of Ns from 3748 to 66. This sequence will permit a much more comprehensive comparison of the ancient and modern VARV genome sequences. Furthermore, the discovery of variant adaptor sequences highlights a serious problem for the assembly of sequencing reads from very old DNA samples when every read counts.

By using the improved assembly of the ancient VARV-VD21 genome together with TATV + CMLV genomes for phylogenetic analysis and the prediction of clade divergence dates, we determined a tMRCA for all VARVs within the range of 1470–1563. This is approximately midway between the latest two estimates from the publications describing the ancient VARV genomes [1,21]. The BEAST software predicted a split of the VARV and CMLV + TATV clades approximately 1250 years prior to the tMRCA for all VARVs. However, such predictions have limited accuracy, and, based on the simple mutation rate of about 40 SNPs per 100 years seen in the core region of the VARV clade, the tMRCA for CMLV + TATV + VARV would be approximately 2000 years before the tMRCA for all VARVs.

An ortholog of the *VACV-Cop-O1L* gene can be found in the vast majority of poxviruses, and its absence from TATV, CMLV, and some attenuated myxoma and vaccinia viruses is notable. When mapping clade specific SNPs previously [16], our attention was attracted to this gene because of the high number of SNPs and their organization, which suggested that recombination events had been responsible for these modifications. With the sequencing of the ancient VARV genome and many new CPXV genomes, including the VARV-like CPXVs, we were able to determine that the introduction of these SNPs (Figure 7) took place before the VARV + TATV + CMLV and the VARV-like CPXVs diverged. Although, as we previously reported, twelve more amino acid changes occurred in the O1L protein after the split of VARVs from CMLV and TATV, this result shows that a closely related precursor of the *VARV-O1L* ortholog is currently circulating in the environment within the VARV-like CPXV population. Therefore, if this protein is indeed associated with the host-adaption of an ancestral VARV to humans, then the ortholog in the VARV-like CPXVs may have some amino acid changes that are associated with adaption to humans [35]. Clearly, this is not an all-or-nothing event; VACVs grow very well in human cells in vitro, as well as in vaccinated humans. In fact, VACV sometimes grows too well in humans and generates adverse vaccine reactions [36]. Similarly, the deletion of the gene is not lethal but is known to affect plaque size [27]. No mammalian ortholog of *O1L* has been identified, but the biological role of the protein appears to act in conjunction with the poxvirus epidermal growth factor-like protein to sustain activation of the mitogen-activated protein kinase kinase/extracellular signal-regulated kinases (MEK/ERK) pathway [28], thereby promoting the survival of the infected cell in the face of apoptotic signals aimed at killing the cell. Similarly, no mechanism has been proposed for this activity, but if protein-protein interactions are involved then it seems likely that relatively minor changes in the amino acid sequence of this protein could modify the protein such that it performs its function more effectively (or less well) in different mammalian species, thereby generating host specificity.

It was also of interest that a drift towards A + T richness could be observed by examining the SNPs in modern VARVs with the ancient genome as a reference. Approximately twice as many G/C to A/T changes than A/T to G/C changes were observed, but, since this drift is taking place in a genome that is already A + T rich (67%), the bias is actually greater. When the genes from A + T and G + C rich poxviruses are compared, not only are there changes to the codon usage for the particular amino acids, but there are also significant changes to the aa composition. For example, isoleucine and arginine are high in A + T rich poxviruses, with respect to alanine and lysine, respectively, whereas the opposite is true in G + C rich poxviruses. Although genus specific analyses of codon use have been performed in the past [37], it is important that the open reading frames (ORFs) used do in fact represent functional genes; otherwise the data is contaminated by non-coding sequences [38]. Some small regions with unusual nucleotide composition are clearly the result of horizontal gene transfer [39,40], whereas the presence/absence of ribonucleotide reductase subunits [41] and a protein associated with the anaphase-promoting complex [42] have more tenuous associations to the control of the overall nucleotide composition of poxvirus genomes. It will be very interesting to re-analyze codon usage with recently sequenced poxviruses since some of these (e.g., kangaroo poxvirus, unpublished) have intermediate A + T richness levels.

## 5. Conclusions

We report a much-improved assembly of a previously published NGS data set for an ancient VARV genome. This improvement was accomplished by modifying the procedure to remove Illumina sequencing tags from the read data and “manually” searching data for reads capable of spanning contig junctions. In addition, we reviewed all regions of the assembly ourselves rather than relying on automation and sequence depth for read support. Using this new assembly with TATV and CMLV genome sequences, we have derived new dates for the tMRCA for the VARV viruses and were able to observe increasing A + T richness in the VARV lineage. Finally, we placed recombination events that modified the VARV ortholog of *VACV-Cop-O1L* and that may have contributed to host range and virulence of VARV prior to the divergence of the VARV-like CPXVs and the VARV + CMLV + TATV clade.

## Figures and Tables

**Table 1:** Orthopox viruses included in phylogenetic analyses.

Species	Strain Name	Year	Country	Host	Accession
CMLV	CMLV-CMS	1970	Iran	Dromedary	AY009089
CPXV (CPXV-like 1)	CPXV-GER_1980_EP4	1980	Germany	Elephant	HQ420895
CPXV (CPXV-like 1)	CPXV-HumBer07/1	2007	Germany	Human	KC813509
CPXV (CPXV-like 1)	CPXV-HumGri07/1	2007	Germany	Human	KC813511
CPXV (CPXV-like 1)	CPXV-HumLan08/1	2008	Germany	Human	KC813492
CPXV (CPXV-like 2)	CPXV-BR	1939	UK	Human	NC_003663
CPXV (CPXV-like 2)	CPXV-FRA_2001_Nancy	2001	France	Human	HQ420894
CPXV (CPXV-like 2)	CPXV-GER_1990_2	1990	Germany	Human	HQ420896
CPXV (CPXV-like 2)	CPXV-GER91	1991	Germany	Human	DQ437593
CPXV (CPXV-like 2)	CPXV-NOR_1994_MAN	1994	Norway	Human	HQ420899
CPXV (Unassigned)	CPXV-GER_1998_2	1998	Germany	Human	HQ420897
CPXV (Unassigned)	CPXV-HumLit08/1	2008	Germany	Human	KC813493
CPXV (VACV-like)	CPXV-FIN_2000_MAN	2000	Finland	Human	HQ420893
CPXV (VACV-like)	CPXV-GRI	1990	Russia	Human	X94355
CPXV (VARV-like)	CPXV-HumAac09/1	2009	Germany	Human	KC813508
ECTV	ECTV-Mos	1947	Russia	Mouse	NC_004105
TATV	TATV-DAH68	1968	Benin	Gerbil	NC_008291
VACV	VACV-Acam3	2003	US	Unknown	AY313848
VACV	VACV-BRA_Serro	2005	Brazil	Unknown	KF179385
VACV	VACV-Cop	1990	US	Unknown	M35027
VACV	VACV-CVA	2007	Germany	Unknown	AM501482
VACV	VACV-IHDW1	2013	Canada	Unknown	KJ125439
VACV	VACV-TT1	2012	China	Unknown	JX489138
VACV	VACV-WR	1982	US	Unknown	NC_006998
VARV	VARV-VD21	2011	Lithuania	Human	KY358055
VARV (major)	VARV-V563	1954	Czech Republic	Human	LT706528
VARV (major)	VARV-BGD75maj	1975	Bangladesh	Human	L22579
VARV (major)	VARV-CHN48	1948	China	Human	DQ437582
VARV (major)	VARV-COG70_46	1970	Congo	Human	DQ437583
VARV (major)	VARV-ETH72_17	1972	Ethiopia	Human	DQ441425
VARV (major)	VARV-GBR44_harv	1944	UK	Human	DQ441444
VARV (major)	VARV-IND64_vel4	1964	India	Human	DQ437585
VARV (major)	VARV-JPN51_hrpr	1951	Japan	Human	DQ441430
VARV (major)	VARV-KOR47	1947	Korea	Human	DQ441432
VARV (major)	VARV-SOM77_1252	1977	Somalia	Human	DQ441438
VARV (major)	VARV-SUM70_222	1970	Sumatra	Human	DQ437591
VARV (major)	VARV-India 1967	1967	India	Human	NC_001611
VARV (minor)	VARV-V1588	1849	Czech Republic	Human	LT706529
VARV (minor)	VARV-SLE68	1969	Sierra Leone	Human	DQ441437
VARV (minor)	VARV-Gar_1966	1966	Brazil	Human	X72086

**Table 2:** Nucleotide changes for SNPs in modern VARVs compared to VD21-uvic.

Starting nt in VARV VD21-Uvic	New nt in Modern Sequence	Major (BGD75maj)	Minor (Gar_1966)
A	C	2	1
	G	35	41
	T	3	5
C	A	9	9
	G	0	2
	T	78	70
G	A	66	64
	C	1	2
	T	4	5
T	A	8	6
	C	36	45
	G	6	2
Total # SNPs		248	252
SNPs that increase A + T%		157	148
SNPs that increase G + C%		79	89

**Table 3:** Nucleotide changes for SNPs in CMLV compared to nucleotides identical in vaccinia virus (VACV), VARV, TATV, and CPXV-HumAac09.

Ancestral	New nt in CMLV Sequence	CMLV
A	C	9
	G	35
	T	12
C	A	54
	G	5
	T	126
G	A	107
	C	7
	T	52
T	A	11
	C	27
	G	12
Total # SNPs		457
SNPs that increase A + T%		339
SNPs that increase G + C%		83

## Supplementary Materials

The following are available online at www.mdpi.com/1999-4915/9/9/253/s1, Gapped FASTA sequence file: MSA of the MAFFT-aligned conserved orthopox core (VACV-Cop F4L-A24R) from CPXV + VARV + VACV + CMLV + TATV genomes. All gaps and Ns have been left intact.

## References

[1] Duggan A.T., Perdomo M.F., Piombino-Mascali D., Marciniak S., Poinar D., Emery M.V., Buchmann J.P., Duchêne S., Jankauskas R., Humphreys M. (2016). 17th century variola virus reveals the recent history of smallpox. Curr. Biol..

[2] Jacobs B.L., Langland J.O., Kibler K.V., Denzler K.L., White S.D., Holechek S.A., Wong S., Huynh T., Baskin C.R. (2009). Vaccinia virus vaccines: Past, present and future. Antivir. Res..

[3] Sánchez-Sampedro L., Perdiguero B., Mejías-Pérez E., García-Arriaza J., di Pilato M., Esteban M. (2015). The evolution of poxvirus vaccines. Viruses.

[4] Thèves C., Crubézy E., Biagini P. (2016). History of smallpox and its spread in human populations. Microbiol. Spectr..

[5] McCollum A.M., Damon I.K. (2014). Human monkeypox. Clin. Infect. Dis..

[6] Esteban D.J., Buller R.M.L. (2005). Ectromelia virus: The causative agent of mousepox. J. Gen. Virol..

[7] Nalca A., Nichols D.K. (2011). Rabbitpox: A model of airborne transmission of smallpox. J. Gen. Virol..

[8] Li G., Chen N., Roper R.L., Feng Z., Hunter A., Danila M.I., Lefkowitz E.J., Buller R.M.L., Upton C. (2005). Complete coding sequences of the rabbitpox virus genome. J. Gen. Virol..

[9] Franke A., Pfaff F., Jenckel M., Hoffmann B., Höper D., Antwerpen M., Meyer H., Beer M., Hoffmann D. (2017). Classification of cowpox viruses into several distinct clades and identification of a novel lineage. Viruses.

[10] Mauldin M., Antwerpen M., Emerson G., Li Y., Zoeller G., Carroll D., Meyer H. (2017). Cowpox virus: What’s in a name?. Viruses.

[11] Dabrowski P.W., Radonić A., Kurth A., Nitsche A. (2013). Genome-wide comparison of cowpox viruses reveals a new clade related to variola virus. PLoS ONE.

[12] Tu S.-L., Nakazawa Y., Gao J., Wilkins K., Gallardo-Romero N., Li Y., Emerson G.L., Carroll D.S., Upton C. (2017). Characterization of eptesipoxvirus, a novel poxvirus from a microchiropteran bat. Virus Genes.

[13] Gershon P.D., Kitching R.P., Hammond J.M., Black D.N. (1989). Poxvirus genetic recombination during natural virus transmission. J. Gen. Virol..

[14] Qin L., Evans D.H. (2014). Genome scale patterns of recombination between co-infecting vaccinia viruses. J. Virol..

[15] Smithson C., Kampman S., Hetman B., Upton C. (2014). Incongruencies in vaccinia virus phylogenetic trees. Computation.

[16] Smithson C., Purdy A., Verster A.J., Upton C. (2014). Prediction of steps in the evolution of variola virus host range. PLoS ONE.

[17] Kazutaka K., Standley D.M. (2013). MAFFT multiple sequence alignment software version 7: Improvements in performance and usability. Mol. Biol. Evol..

[18] Hillary W., Lin S.-H., Upton C. (2011). Base-by-base version 2: Single nucleotide-level analysis of whole viral genome alignments. Microb. Inform. Exp..

[19] Stamatakis A. (2014). RAxML version 8: A tool for phylogenetic analysis and post-analysis of large phylogenies. Bioinformatics.

[20] Bouckaert R., Heled J., Kühnert D., Vaughan T., Wu C.-H., Xie D., Suchard M.A., Rambaut A., Drummond A.J. (2014). BEAST 2: A software platform for Bayesian evolutionary analysis. PLoS Comput. Biol..

[21] Pajer P., Dresler J., Kabíckova H., Písa L., Aganov P., Fucik K., Elleder D., Hron T., Kuzelka V., Velemínsky P. (2017). Characterization of two historic smallpox specimens from a Czech museum. Viruses.

[22] Martin M. (2011). Cutadapt removes adapter sequences from high-throughput sequencing reads. EMBnet. J..

[23] Chevreux B., Pfisterer T., Drescher B., Driesel A.J., Müller W.E.G., Wetter T., Suhai S. (2004). Using the miraEST assembler for reliable and automated mRNA transcript assembly and SNP detection in sequenced ESTs. Genome Res..

[24] Bankevich A., Nurk S., Antipov D., Gurevich A.A., Dvorkin M., Kulikov A.S., Lesin V.M., Nikolenko S.I., Pham S., Prjibelski A.D. (2012). SPAdes: A new genome assembly algorithm and its applications to single-cell sequencing. J. Comput. Biol..

[25] Milne I., Stephen G., Bayer M., Cock P.J.A., Pritchard L., Cardle L., Shaw P.D., Marshall D. (2013). Using tablet for visual exploration of second-generation sequencing data. Brief. Bioinform..

[26] Li Y., Carroll D.S., Gardner S.N., Walsh M.C., Vitalis E.A., Damon I.K. (2007). On the origin of smallpox: Correlating variola phylogenics with historical smallpox records. Proc. Natl. Acad. Sci. USA.

[27] Schweneker M., Lukassen S., Späth M., Wolferstätter M., Babel E., Brinkmann K., Wielert U., Chaplin P., Suter M., Hausmann J. (2012). The vaccinia virus O1 protein is required for sustained activation of extracellular signal-regulated kinase 1/2 and promotes viral virulence. J. Virol..

[28] Bonjardim C.A. (2017). Viral exploitation of the MEK/ERK pathway—A tale of vaccinia virus and other viruses. Virology.

[29] Martin D.P., Murrell B., Golden M., Khoosal A., Muhire B. (2015). RDP4: Detection and analysis of recombination patterns in virus genomes. Virus Evol..

[30] Senkevich T.G., Koonin E.V., Bugert J.J., Darai G., Moss B. (1997). The genome of molluscum contagiosum virus: Analysis and comparison with other poxviruses. Virology.

[31] Lakis N.S., Li Y., Abraham J.L., Upton C., Blair D.C., Smith S., Zhao H., Damon I.K. (2015). Novel poxvirus infection in an immune suppressed patient. Clin. Infect. Dis..

[32] Miranda J.B., Borges I.A., Campos S.P.S., Vieira F.N., de Ázara T.M.F., Marques F.A., Costa G.B., Luis A.P.M.F., de Oliveira J.S., Ferreira P.C.P. (2017). Serologic and molecular evidence of vaccinia virus circulation among small mammals from different biomes, Brazil. Emerg. Infect. Dis..

[33] Fleming S., Wise L., Mercer A. (2015). Molecular genetic analysis of orf virus: A poxvirus that has adapted to skin. Viruses.

[34] Springer Y.P., Hsu C.H., Werle Z.R., Olson L.E., Cooper M.P., Castrodale L.J., Fowler N., McCollum A.M., Goldsmith C.S., Emerson G.L. (2017). Novel orthopoxvirus infection in an Alaska resident. Clin. Infect. Dis..

[35] Sawyer S.L., Elde N.C. (2012). A cross-species view on viruses. Curr. Opin. Virol..

[36] Vellozzi C., Lane J.M., Averhoff F., Maurer T., Norton S., Damon I., Casey C. (2005). Generalized vaccinia, progressive vaccinia, and eczema vaccinatum are rare following smallpox (vaccinia) vaccination: United States surveillance, 2003. Clin. Infect. Dis..

[37] Roy Choudhury S., Pan A., Mukherjee D. (2011). Genus specific evolution of codon usage and nucleotide compositional traits of poxviruses. Virus Genes.

[38] Da Silva M., Upton C. (2005). Using purine skews to predict genes in AT-rich poxviruses. BMC Genom..

[39] Da Silva M., Upton C. (2005). Host-derived pathogenicity islands in poxviruses. Virol. J..

[40] Monier A., Claverie J.-M., Ogata H. (2007). Horizontal gene transfer and nucleotide compositional anomaly in large DNA viruses. BMC Genom..

[41] Gammon D.B., Gowrishankar B., Duraffour S., Andrei G., Upton C., Evans D.H. (2010). Vaccinia virus-encoded ribonucleotide reductase subunits are differentially required for replication and pathogenesis. PLoS Pathog..

[42] Mo M., Fleming S.B., Mercer A.A. (2010). Orf virus cell cycle regulator, PACR, competes with subunit 11 of the anaphase promoting complex for incorporation into the complex. J. Gen. Virol..

